# Blood Eosinophilia Is an on-Treatment Biomarker in Patients with Solid Tumors Undergoing Dendritic Cell Vaccination with Autologous Tumor-RNA

**DOI:** 10.3390/pharmaceutics12030210

**Published:** 2020-03-01

**Authors:** Alvaro Moreira, Michael Erdmann, Ugur Uslu, Verona Vass, Gerold Schuler, Beatrice Schuler-Thurner

**Affiliations:** Department of Dermatology, University Hospital Erlangen, Friedrich-Alexander-University Erlangen-Nürnberg (FAU), 91054 Erlangen, Germany; michael.erdmann@uk-erlangen.de (M.E.); ugur.uslu@uk-erlangen.de (U.U.); verona-bernadette.vass@uk-erlangen.de (V.V.); gerold.schuler@uk-erlangen.de (G.S.); beatrice.schuler-thurner@uk-erlangen.de (B.S.-T.)

**Keywords:** dendritic cell vaccines, biomarkers, eosinophils

## Abstract

Background: The approvals of immune checkpoint inhibitors for several cancer types and the rapidly growing recognition that T cell-based immunotherapy significantly improves outcomes for cancer patients led to a re-emergence of cancer vaccines, including dendritic cell (DC)-based immunotherapy. Blood and tissue biomarkers to identify responders and long-term survivors and to optimize cost and cost-effectiveness of treatment are greatly needed. We wanted to investigate whether blood eosinophilia is a predictive biomarker for patients with solid tumors receiving vaccinations with DCs loaded with autologous tumor-RNA. Methods: In total, 67 patients with metastatic solid tumors, who we treated with autologous monocyte-derived DCs transfected with total tumor mRNA, were serially analyzed for eosinophil counts and survival over the course of up to 14 years. Eosinophilic counts were performed on peripheral blood smears. Results: Up to 87% of the patients treated with DC-based immunotherapy experienced at least once an eosinophilia of ≥ 5% after initiation of therapy; 61 % reached levels of ≥ 10% eosinophils, and 13% of patients showed eosinophil counts of 20% or above. While prevaccination eosinophil levels were not associated with survival, patients with blood eosinophilia at any point after initiation of DC-based immunotherapy showed a trend towards longer survival. There was a statistically significant difference for the patients with eosinophil counts of 20% or more (p = 0.03). In those patients, survival was prolonged to a median of 58 months (range 2–111 months), compared to a median of 20 months (range 0–119 months) in patients with lower eosinophil counts. In 12% of the patients, an immediate increase in eosinophil count of at least 10 percentage points could be detected after the first vaccine, which also appeared to correlate with survival (65 vs. 24 months; p = 0.06). Conclusion: Blood eosinophilia appears to be an early, on-therapy biomarker in patients with solid tumors undergoing vaccination with RNA-transfected DC, specifically autologous tumor mRNA-transfected DC vaccines, and it correlates with long-term patient outcome. Eosinophilia should be systematically investigated in future trials.

## 1. Background

The approvals of immune checkpoint inhibitors for several cancer types and the rapidly growing recognition that immunotherapy significantly improves outcomes for cancer patients led to a re-emergence of cancer vaccines, including dentritic cell (DC)-based immunotherapy [1,2,3,4,5,6,7]. DCs are essential in immunity due to their role in priming, activating, and directing the T cells to target tumor cells and, thereby, by promoting antitumorigenic responses [1,8,9]. While tumor-induced T cell exhaustion may be partially reversed by checkpoint inhibitors, current treatments fail to show clinical benefits in a significant portion of the patients. With novel techniques of DC generation being developed [2,10], and combination regimes with other therapies, such as checkpoint inhibitors, being evaluated in preclinical and clinical settings [11,12,13,14], DC vaccination is still viewed as an opportunity to foster immunity against tumors [15,16,17,18].

Blood and tissue biomarkers to identify responders and long-term survivors and to optimize cost and cost-effectiveness of treatment are greatly needed. Eosinophilia has been associated with a better outcome for DC vaccination (reported for the Provenge™ vaccine [19,20], for our peptide-loaded DCs [21]), as well as for checkpoint inhibitors, including ipilimumab [22,23,24] and pembrolizumab [24]. In metastatic melanoma, prevaccination eosinophilic count has been shown to be a prognostic marker, independently of the subsequent treatment [25].

Eosinophils have a unique contribution in initiating immune responses, due to their bidirectional interactions with dendritic cells and T cells [26]. Studies have suggested eosinophils to be part of an early inflammatory reaction at the site of tumorigenesis [27]. Eosinophils are not only a source of numerous cytokines and growth factors, but they also display both proinflammatory and anti-inflammatory activities, as well as immunoregulatory ones, which are most likely regulated by a complex network determined by surface molecules, extracellular components, and cell–cell interactions [28,29,30,31,32,33]. The role of eosinophils in allergic diseases led to several studies of the association between allergic conditions and cancer incidence [34,35]. Interestingly, these reported an inverse relationship between allergy-based diseases and several malignancies, including lung cancer, colorectal cancer, prostate cancer, breast cancer, and pancreatic cancer [36,37,38,39,40,41,42,43,44]. As most of the studies neither assessed confounding factors at the patient level nor incorporated them into the analysis, the association between allergic diseases accompanied by eosinophilia and cancer risk remains conflicting [45]. Clinical studies of adoptive T-cell therapy in patients with metastatic melanoma showed an increase in eosinophils to up to 50% in over 60% of the patients [46], which appears to result from IL-2 administration [47,48].

Transient eosinophilia occurred after dendritic cell vaccination in men with metastatic prostate cancer treated with Provenge™ [19,20], and was proposed to be an indicator of global immune activation and survival benefit. Whether this constitutes just a paraneoplastic epiphenomenon, as it has been suggested by some authors [49], is still to be determined. Similar to the initial transient eosinophilia, tissue studies have also shown that the infiltration of solid tumors by eosinophils is an early immunological response [27]. Conversely, eosinophilic infiltration is considered unfavorable in hematologic malignancies. Eosinophils are recruited to tumor tissues and interact with tumor cells, through secreting granule protein and cytokines, or by presenting tumor antigens to activate other types of immune cells, which might be part of the process of its antitumor mechanism [45].

In this study, we wanted to investigate whether eosinophilia is a biomarker for patients with solid tumors receiving vaccinations with DCs loaded with RNA, specifically autologous tumor-RNA.

## 2. Methods

**Figure 1:**
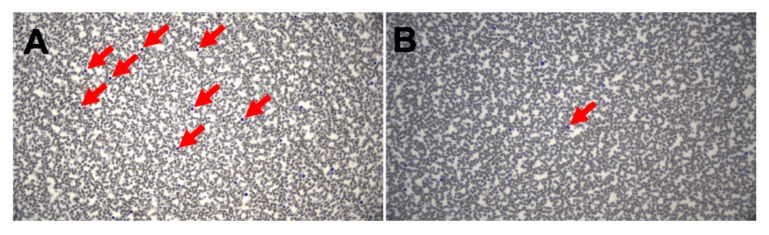
Representative images of patients with (**A**) higher and (**B**) lower number of eosinophils in blood smears (original magnification 400×).

In total, 67 patients with metastatic solid tumors (*n* = 34 with cutaneous melanoma; *n* = 13 with uveal melanoma; *n* = 4 with colon cancer; *n* = 3 with neuroendocrine tumors; *n* = 3 with mucosal melanoma; *n* = 2 with urothelial carcinoma; *n* = 2 with renal cell carcinoma; *n* = 1 with glioblastoma; *n* = 1 with pleomorphic xanthoastrocytoma; *n* = 1 with ovarian cancer; *n* = 1 with pancreatic cancer; *n* = 1 with prostate cancer; and *n* = 1 with leiomyosarcoma), who received autologous monocyte-derived DCs transfected with autologous tumor mRNA, were serially analyzed for eosinophil counts and survival over the course of up to 14 years. Standardized, mature, monocyte-derived DCs were generated from apheresis as described [50], and then electroporated with PCR-amplified autologous total tumor mRNA as described [51,52]. DCs were administered intravenously and/or intradermally. Eosinophilic count was detected by peripheral blood smear (Figure 1). We performed an intention-to-treat (ITT) analysis with all 67 patients and a per-protocol (PP) analysis with 41 patients who had a blood draw after the first vaccine. From the other 26 patients, there was no differential blood test performed after the first vaccine, although almost all of them (*n* = 66) received more than one vaccine. Gehan–Breslow–Wilcoxon tests were performed to determine the p-value. Graphing and statistics were performed using GraphPad Prism.

This study was exempt from full application to the Ethics Committee, University of Erlangen. (references numbers 1341 and 12_2011). Datasets used and analyzed during the study are available from the corresponding author upon reasonable request.

## 3. Results

**Figure 2:**
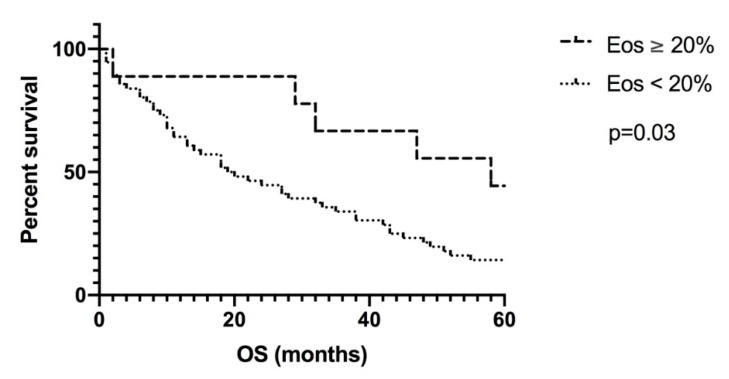
Intention-to-treat analysis for a cut-off of 20% eosinophils in peripheral blood. DC-vaccinated patients who developed eosinophilia ≥ 20% at any point during the course of vaccinations showed a clear trend toward longer survival (*p* = 0.03). In these patients, survival was prolonged with a median of 58 months (range 2–111 months; *n* = 9), compared with a median of 20 months (range 0–119 months; *n* = 58).Eos: eosinophilia; OS: overall survival.

Our findings revealed that a large percentage of patients with metastatic solid tumors develop eosinophilia during or after initiation of the DC-based immunotherapy. We defined eosinophilia as a percentage of at least 5% eosinophils in peripheral blood and analyzed survival for that value, but also for cut-offs of 10% and 20% eosinophils. In the ITT analysis of the 67 patients treated with DC-based immunotherapy, 87% of them experienced at least once an eosinophilia greater than or equal to 57% after initiation of DC-immunotherapy, 61% reached levels of at least 10% eosinophils, and 13% of patients showed eosinophil counts of 20% and above. DC-vaccinated patients who developed eosinophilia of 20% or more at any point during the course of vaccinations showed a clear trend toward longer survival (*p* = 0.03; Figure 2). In those patients, survival was prolonged with a median of 58 months (range 2–111 months; *n* = 9), compared with a median of 20 months (range 0–119 months; *n* = 58) in patients with lower eosinophil counts.

**Figure 3:**
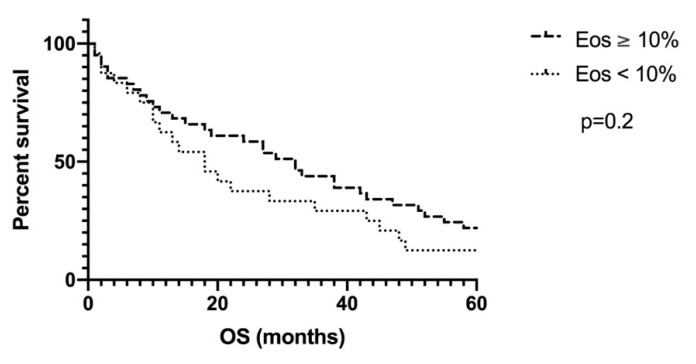
Intention-to-treat analysis for a cut-off of 10% eosinophils in peripheral blood. With a cut-off at a percentage of eosinophils of 10%, the median OS for patients with eosinophilia greater than 10% (*n* = 41) was 32 months, compared with 18 months for patients with less than 10% eosinophils (*n* = 26). Eos: eosinophilia; OS: overall survival.

The median overall survival (OS) was 28 months for patients with a percentage of eosinophils higher than 5% (*n* = 58), compared with 13 months for patients with a percentage of eosinophils less than 5% (*n* = 9). With a cut-off at a percentage of eosinophils of 10%, the median OS for patients with eosinophilia greater than 10% (*n* = 41) was 32 months, compared with 18 months for patients with less than 10% eosinophils (*n* = 26; Figure 3), although this was not statistically significant (*p* = 0.2), possibly also due to the size and the heterogeneity of the cohorts.

**Figure 4:**
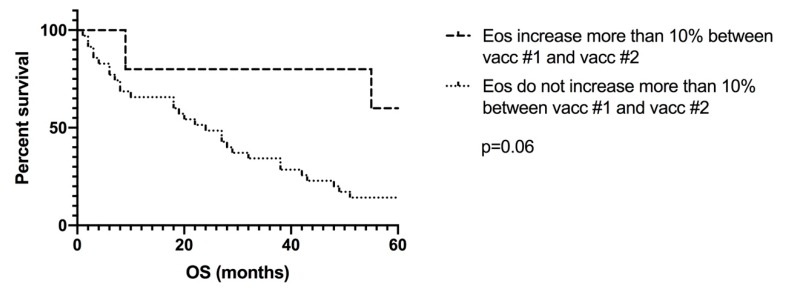
Per-protocol analysis to evaluate the early change in eosinophil count between the first and the second DC vaccines. In 12% of the patients (*n* = 5), an immediate increase in eosinophil count of at least 10 percentage points could be detected after the first vaccine, which appeared to correlate with survival (65 vs. 24 months; *p* = 0.06). DC: dendritic cells; Eos: eosinophilia; OS: overall survival.

With the PP analysis, we could evaluate the early change in eosinophil count between the first and the second DC vaccines. Interestingly, in 12% of the patients (*n* = 5) an immediate increase in eosinophil count of at least 10 percentage points could be detected after the first vaccine, which appeared to correlate with survival (65 vs. 24 months; *p* = 0.06; Figure 4). This trend could also be seen for an increase in eosinophil count of at least 5 percentage points after the first DC vaccine (53 vs. 25 months).

**Figure 5:**
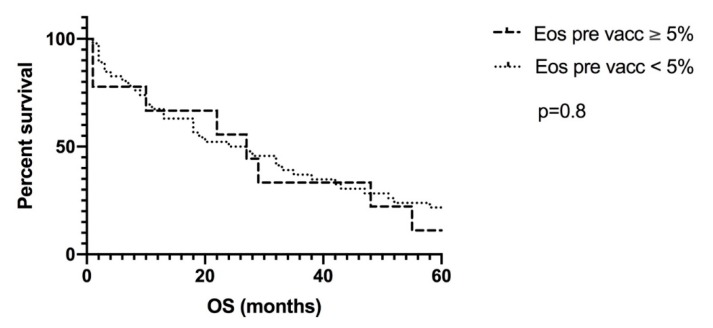
Analysis of the prevaccination eosinophil counts with OS. Prevaccination eosinophil levels did not associate with better outcome. There were nine patients with a preexisting eosinophilia of 5% or more, compared to 48 patients with preexisting values of less than 5%. Eos: eosinophilia; OS: overall survival.

In order to assess whether the favorable outcome could be independent from DC vaccination, we analyzed prevaccination eosinophil counts, which were available for 57 patients, with OS. Prevaccination eosinophil levels clearly did not associate with survival (Figure 5).

## 4. Discussion

Our results suggest that a very early increase of eosinophils upon start of DC vaccination has predictive value for long-term outcome. This phenomenon is also reminiscent of our observation that early post-vaccination increase in PEBP1-mRNA in blood correlates with survival [21]. Our study demonstrates that RNA-transfected DCs (in this case DCs transfected by autologous tumor mRNA) induce blood eosinophilia, similar to what has previously been described for peptide/protein-loaded DC vaccines [19]. Therefore, this phenomenon does not appear to be dependent on the nature of the vaccine antigens involved, but rather on using DC as vaccine vectors. 

There are limitations in the present study, due to the size of the cohort and the heterogeneity of the cancer types. For this reason, we recommend the systematic evaluation of eosinophilia as potential biomarker, including prospectively, in larger cancer immunotherapy trials.

In addition, our understanding of the mechanism leading to the induction of eosinophilia and underlying the predictive role of eosinophils in DC vaccinated patients will require further fundamental and translational studies. Indeed, the current data cannot yet be reconciled into a concise picture.

A role for eosinophils as antigen-presenting cells (APC) was proposed several years ago [53]. More recently, evidence has indicated that the APC-like phenotype of eosinophils is influenced by NK-cells [29]. Eosinophils functionally interact with NK cells during the early phases of innate immune responses, participating in immune crosstalk. They further improve NK cell’s ability to induce DC editing and maturation [29]. Additional mechanistic studies will be critical to the understanding of the role played by eosinophils in cancer and vaccination. These should include studies on the functional aspects of eosinophils and eosinophil granule proteins, such as eosinophil cationic protein (ECP), eosinophil peroxidase (EPO) and eosinophil-derived neurotoxin (EDN). Eosinophilic serum biomarkers, such as ECP, have already been studied in the context of metastatic disease and appear to be of prognostic value [54] in metastatic melanoma.

Future challenges will address the need for the identification of novel surface markers for eosinophil detection and the current lack of studies involving eosinophils within the tumor microenvironment [55]. Furthermore, classifying eosinophils into subpopulations based on their secretome, cytokine, and MHC class expression will help one to decipher the complexity of this cell type [55].

Future therapies could benefit from specifically targeting eosinophils [45,56,57,58] in order to optimize tumor rejection. Indeed, induction of eosinophils does not appear to simply constitute a bystander phenomenon but seems to directly or indirectly mediate tumor control [59,60,61,62,63,64,65]. 

Finally, eosinophils are particularly abundant in the lamina propria of the gastrointestinal tract. Considering the growing role of microbiota in the immune response to cancer [66], it is reasonable to speculate that there might be a role for microbiome-directed therapy to target and modulate eosinophils.

## 5. Conclusions

In our study, blood eosinophilia correlates positively with the survival of patients with solid tumors undergoing vaccination with tumor mRNA-transfected DCs. This association is particularly noticeable after an early and strong increase in eosinophil count (≥ 10 percentage points) upon initial DC vaccination. Furthermore, patients who developed blood eosinophilia at any point after initiation of vaccination showed a trend toward longer survival. This association is stronger for patients reaching eosinophilia levels of ≥ 20%.

Previous reports have shown that peptide-/protein-loaded DC vaccines induce eosinophilia, and that this parameter is a prognostic marker. The present study demonstrates that RNA-transfected DCs, specifically autologous tumor mRNA transfected DC vaccines, also induce eosinophilia, and that this correlates with survival. Thus, this phenomenon is neither dependent on the nature of vaccine antigens nor the loading strategy involved, but inherent to using DC as vaccine vectors. This novel finding is in line with reports that the development of blood eosinophilia upon treatment with immunotherapy drugs, such as checkpoint inhibitors anti-CTLA-4 and anti-PD-1, is a predictor of the clinical response.

Measurement of eosinophilic counts should, therefore, be routinely included as a potential prognostic marker for DC vaccination, notably in prospective clinical trials. Furthermore, the functional aspects of eosinophils should continue to be evaluated.
